# Changes in Grandparenting During the Pandemic and Effects on Mental Health: Evidence From England

**DOI:** 10.1093/geroni/igab046.1240

**Published:** 2021-12-17

**Authors:** Giorgio Di Gessa, Valeria Bordone, Bruno Arpino

**Affiliations:** 1 University College London, London, England, United Kingdom; 2 University of Vienna, Vienna, Wien, Austria; 3 University of Florence, Florence, Toscana, Italy

## Abstract

Policies aiming at reducing rates of hospitalisation and death from Covid-19 encouraged older people to reduce their physical contacts. For grandparents in England, this meant that provision of care for grandchildren was allowed only under very limited circumstances. To date, evidence on changes in grandparenting during the pandemic is scarce and little is known about whether and to what extent reduction in grandchild care provision impacted grandparents’ mental health. Using pre-pandemic data from Wave 9 (2018/19) and the second Covid-19 sub-study (November/December 2020) of the English Longitudinal Study of Ageing, we first described changes in grandparenting since the start of the pandemic. Then, using regression models, we investigated associations between changes in grandparenting and mental health (depression, quality of life, life satisfaction, and anxiety) during the pandemic, while controlling for pre-pandemic levels of the outcome variables. Almost a third of grandparents reported that the amount of grandchild care during the pandemic reduced or stopped altogether, whereas 10% provided as much or more care compared to pre-pandemic levels, mostly to help parents while working. Compared to grandparents who provided grandchild care at some point during the pandemic, those who stopped altogether were more likely to report poorer mental health, even taking into account pre-pandemic health. A reduction in grandparenting was only marginally associated with higher depression. Although policies to limit physical contacts and shield older people reduced their risks of getting ill from Covid-19, our study shows the consequences of stopping childcare provision in terms of poorer mental health among grandparents.

